# Microsatellite analysis for differentiating the origin of renal angiomyolipoma and involved regional lymph node

**DOI:** 10.1038/s41598-017-00460-w

**Published:** 2017-03-23

**Authors:** Ping Tan, Huan Xu, Yong Jiang, Lu Yang, Yan Zou, Liangren Liu, Nian Liu, Dehong Cao, Yu Fan, Qiyuan Li, Qiang Wei

**Affiliations:** 1 Department of Pathology, West China Hospital, Sichuan University, Chengdu, Sichuan People’s Republic of China; 2 Department of Urology, West China Hospital, Sichuan University, Chengdu, Sichuan People’s Republic of China; 3 Institute of Urology, West China Hospital, Sichuan University, Chengdu, Sichuan People’s Republic of China

## Abstract

Renal angiomyolipoma (AML) with the regional lymph node (LN) involved leads to a question of metastasis versus multicentric origin when their histology are similar. As the genomic instability is a common feature of cancer, we hypothesized that independently arising neoplasms in an individual patient would exhibit measurable genomic variation, facilitating the discrimination of tumor lineage and relatedness. Our study enrolled 12 patients who were diagnosed with nodal-involved renal AML at West China Hospital. Genomic DNA from kidney and lymph node lesion from individuals was analyzed through PCR-based analysis which using six microsatellite markers to identify discordant allelic variation. The results showed all 12 patients underwent surgical treatment and none suffered local recurrence or distant metastasis during the follow-up. Ten patients of the included cases showed a consistent trend that all corresponding to six microsatellite markers were detected in DNA from renal AMLs but were reduced or not observed in DNA from the paired LN. With this technique, a possible lineage relationship cannot be excluded between renal AMLs and LN. Thus when enlarged LN were found in images, active surveillance should be taken into consider; if enlarged LN were found intraoperatively, LN resection might be necessary to demonstrate their pathological nature.

## Introduction

Renal angiomyolipoma (AML), a non-aggressive benign tumor, occurs either sporadically or in association with tuberous sclerosis complex. AML is composed of mature adipose tissue, smooth muscle cells and thick-walled blood vessels. Typically, it displays a slow growth pattern and lacks distant metastasis. However, its locally aggressive feature has been reported^1^. Moreover, AML-derived cells have mutations in one of the two TSC genes (TSC-1 and TSC-2), especially TSC-2^2^. Over the decades, more than 40 cases of renal AML with nodal involvement have been reported, suggesting the metastatic potential of AML^3^. However, due to its benign nature and absence of recurrence or distant metastasis, it was claimed that this nodal involvement was just a multicentric growth pattern rather than metastasis^4–6^.

Despite well-established histological features and sophisticated immunostaining techniques, primary neoplasm and its involved lymph nodes (LNs) are still difficult to distinguish due to similar histological features. Fortunately, genomic instability, an intriguing hallmark of neoplasm, differs among independently developing neoplasms and can be used to generate unique neoplasm signatures that reflects tumor lineage and relatedness. These genetic alterations can be detected by polymerase chain reaction (PCR)-based microsatellite analysis, by DNA sequencing, and occasionally by immunohistochemistry^7,8^.

Therefore, in this study, we aimed to analyze the genomic instability of 12 patients with nodal-involved renal AML and to determine the lineage relationship between renal AML and involved lymph nodes through a PCR-based microsatellite analysis.

## Material and Methods

### Patients and clinical features

All candidates came from the West China Hospital, a high-volume clinical care center. The key word “AML” (in Chinese) were used to search patients who underwent a surgical resection between January 2000 and June 2015 in Pathology Database. Total 1622 cases of AML were retrieved, including 1272 renal AML patients. After screening, we found 71 patients (71/1272 = 5.6%) who had regional lymph nodes enlargement. Their paraffin-embedded tissue slides were then reviewed by two senior pathologists. Finally, 12 (12/1272 = 0.9%; 12/71 = 16.9%) renal AML patients with regional LNs involved were included. The LNs from remaining 59 cases were all diagnosed with reactive hyperplasia and then excluded. In each individual patient, only a single LN was involved, the remaining evaluated LNs were diagnosed with reactive hyperplasia. The study was approved by the Ethics Committee of West China Hospital and the methods were carried out in accordance with the approved guidelines. By phone calls, oral informed consent was obtained for reviewing and analyzing their pathological specimens. The hospitalization and re-examination records of all eligible patients were then reviewed, including basic features, images, types of treatment, etc.. All patients were suggested yearly chest X-ray for three years and imaging at yearly intervals for three years with abdominal US, CT or MRI^9^. Considering the benign nature of typical renal AML, these imaging examinations were voluntary choices after three years. Participants were followed up yearly by making phone calls or in out-patient department of urology.

### Macrodissection and DNA preparation

For each patient, consecutive sections (5 µm) were cut from formalin-fixed and paraffin-embedded tumor tissues. One of the sections was stained with H & E staining and regions of interest were marked on that section, which was used as a reference for macrodissection. Then, tumor tissues in the regions of interest were dissected from 6–10 unstained sections and deparaffinzed in xylene. After several cycles of centrifuging and pellet harvesting, the final pellets were obtained and processed for DNA extractions. DNA extractions were performed by using QIAGEN QIAamp® DNA FFPE Tissue Kit (QIAGEN GmbH, 56404, Germany) according to the manufacturer’s instructions. The DNA concentration of each sample was measured by ScanDrop100 (Analytic Jena, Germany).

### Microsatellite PCR-based analysis

Genomic DNA collected from tumor samples were examined for 6 polymorphic microsatellite markers with high reproducibility in a PCR-based assay of AML, including: RH68414, SHGC-30026, csnpnthl1-pcr1–1, GDB:378419, STS-L48546, and D16S3394. Information concerning these markers and primer sequences is available on the Genome Database (http://www.gdb.org/) and the NCBI genome database (http://www.ncbi.nlm.nih.gov/). The oligonucleotide primers corresponding to each microsatellite marker (MapPairs™primers) were purchased from INVITROGEN TRADING (SHANGHAI) CO. LTD. PCR-based amplification was then performed. PCR products were fractioned on 10% polyacrylamide gels containing 0.5 M EDTA (PH 8.0), stained by silver nitrate and imaged by ChemiDoc MP (BIORAD). PCR reactions that did not produce detectable amplified products were re-amplified to confirm a negative result.

## Results

**Figure 1:**
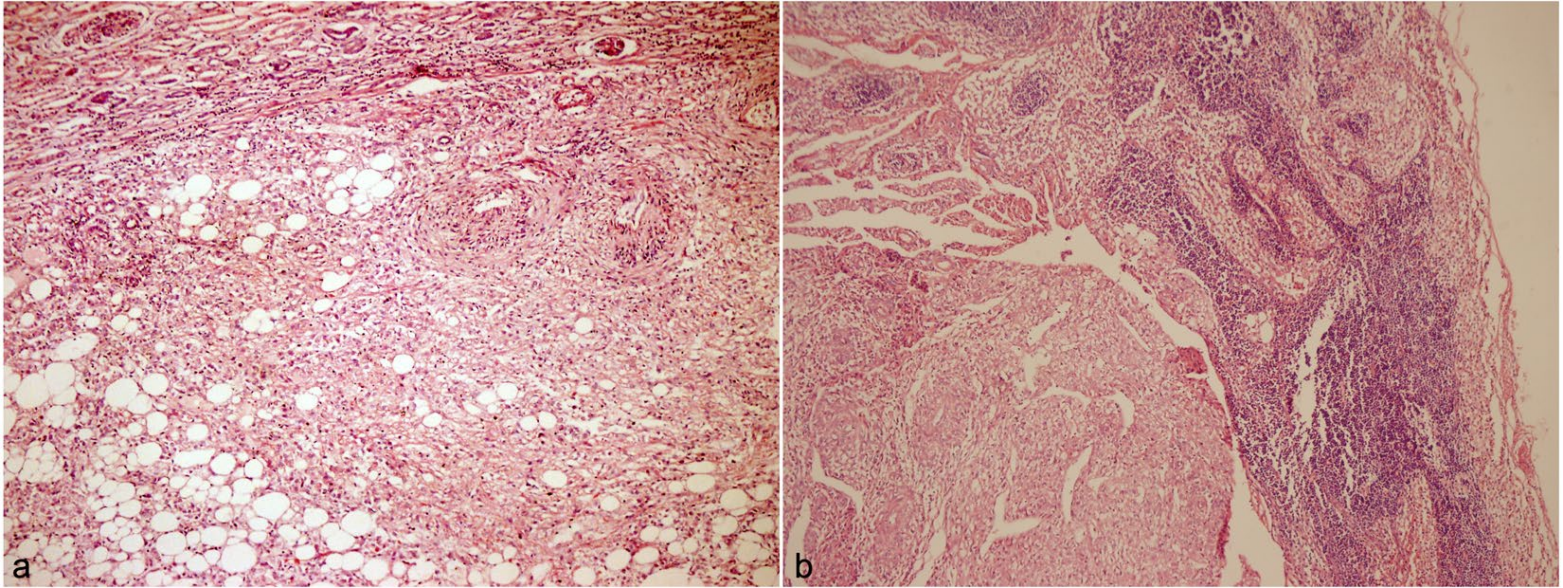
HE staining of Patient 9. (**a**) (Left), the renal angiomyolipoma; (**b**) (Right), the angiomyolipoma in lymph node. HE × 100.

**Figure 2:**
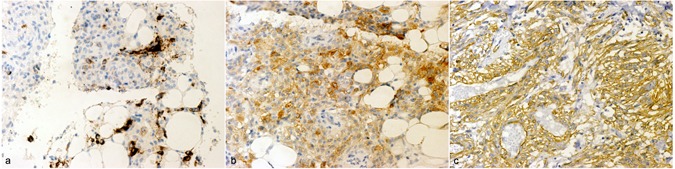
IHC staining of Patient 9. (**a**) (Left), the tumor cells show focal positive for HMB-45; (**b**) (Middle), the tumor cells show focal positive for MART-1; (**c**) (Right), the tumor cells show strong positive for SMA. LsAB × 400.

Of the 12 patients enrolled, two were unavailable to determine the survival status because of the invalid phone numbers. Finally, 10 patients (83.3%) completed the follow-up, with a mean follow-up duration of 80.4 months. 11 cases of included patients were female, and only one male. Two adolescents (16.7%) were found in the present study. One of them (8.3%) had bilateral tumors without family history or TSC and underwent nephron-sparing surgery, and another one had the largest tumor size and was treated with radical nephrectomy. All follow-up patients were alive and none suffered local recurrence or distant metastasis. No family history was found among all cases. HMB-45 and SMA were positive in 100% and 91.7% of patients, respectively. Seven of twelve Patients (58.3%) included in present study showed no symptom and were discovered with imaging. No hematuria was observed in all patients. The clinical data and pathological features of all 12 patients were showed in Table 1. The histological features of primary renal AML was similar to that of involved node in each individual patient (Fig. 1). Figure 2 showed a part of Immunohistochemistry staining results.

**Table 1:** Summary of clinical and pathological data of patients diagnosed with renal AML and involved regional lymph node.

**IHC stain**	
HMB-45	12/12(100%, +)
SMA	11/12(91.7%, +); 1/12(8.3%, unclear)
MART-1	2/12(16.7%, +); 1/12(8.3%, −); 9/12(75%, unclear)
Desmin	2/12(16.7%, +); 3/12(25%, −); 7/12(58.3%, unclear)
S100	4/12(33.3%, −); 8/12 (66.7%, unclear)
Ki-67	3/12[25%, (+, <2%)]; 1/12[8.3%,(+, <10%)]; 8/12(66.7%, unclear)
**Follow-up data**	
Follow-up duration/month	Mean: 80.4 (8–180)
Survival status	alive: n = 10 (83.3%); unclear: n = 2 (16.7%)
Recurrence	n = 0
Metastasis	n = 0
**Baseline data**	
Gender	Female/Male = 11/1
Mean age/years	38.5 (10–65)
Associated symptoms/signs	backache: n = 5 (41.7%); kidney area kowtow painful: n = 1 (8.3%); without: n = 7 (58.3%)
kidney	Confined to kidney: n = 5 (41.7%); Beyond kidney: n = 7 (58.3%)
Position	Upper pole of kidney: n = 8; multiple: n = 1; middle part of kidney: n = 2; retroperitoneal: n = 1
Size/cm^3^	Mean: 924.2(55.9–6300); Max: 35*15*12 cm (Patient 12)
Lymph nodal	renal hilus node: n = 6 (50%); para-aortic node: n = 4 (33.3%); para- arteriorenal node: n = 1 (8.3%); venacaval node: n = 1 (8.3%)
Surgical method	radical nephrectomy: n = 10 (83.3%); nephron-sparing surgery: n = 1 (8.3%); radical nephrectomy + Adrenalectomy: n = 1 (8.3%)

### Molecular analysis of primary renal tumor and involved lymph node with similar histological types

**Figure 3:**
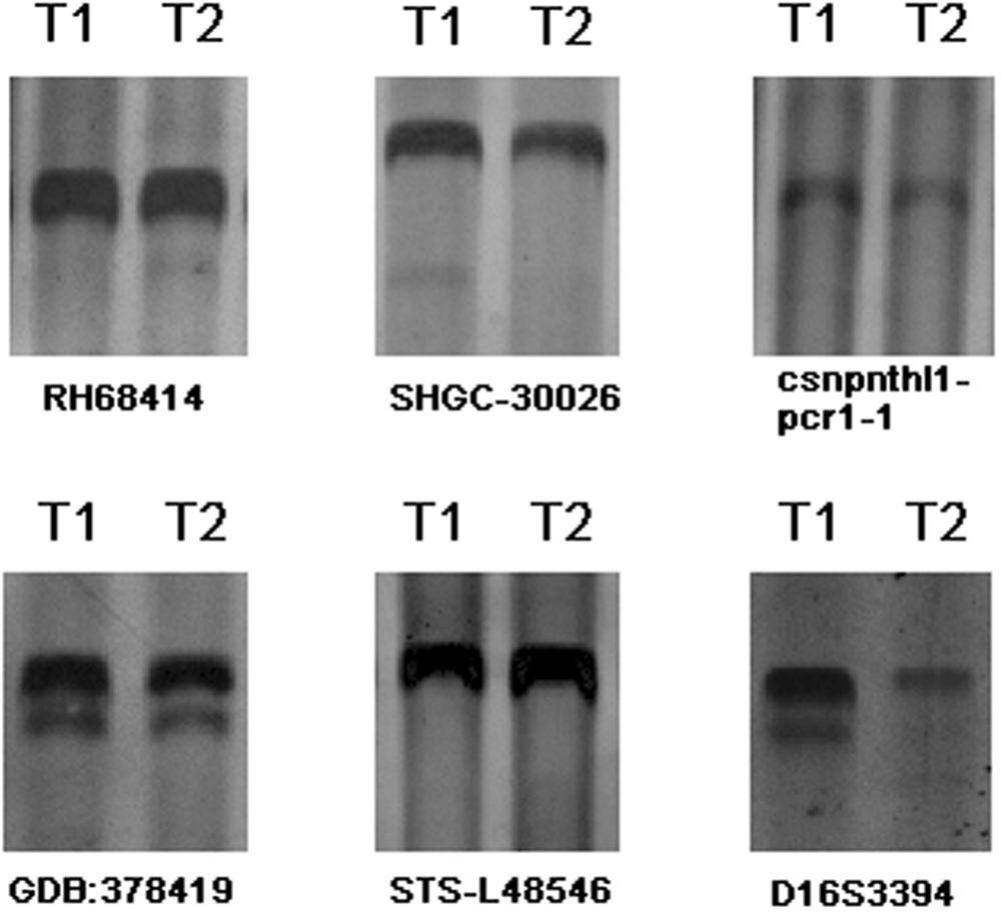
Molecular analysis of Patient 9. T1 refers to the AML in kidney; T2 refers to the paired involved lymph node. The identity of each microsatellite marker analyzed is indicated. (10% polyacrylamide gels containing 0.5 M EDTA (PH 8.0), stained by silver nitrate; gels have been run under the same experimental conditions).

Molecular signatures were generated for each of 24 samples (12 samples from kidneys; 12 samples from the paired nodes) using six polymorphic microsatellite markers. All PCR results were shown in Table 2. The six microsatellite markers examined identified allelic variation in 8.3% (1/12) to 33.3% (4/12) of patients. Figure 3 showed a representative result from microsatellite PCR analysis of tumors corresponding to Patient nine. T1 refers to AML in kidney and T2 refers to AML in LN. In the case of Patient one, T1 and T2 shared the same allelic patterns in four microsatellite markers, but showed differing allelic patterns in two microsatellite markers at Csnpnthl1-pcr1–1 and GDB: 378419 (see Table 2). The pattern of allelic variation presented allelic loss in these two microsatellite markers direction in T2 of this patient, which suggested that T2 could be derived from T1. Similar results were also obtained in other four patients (Patient two, four, nine, and twelve). In other five patients (Patient three, five, seven, eight, and eleven), only one allelic variation was observed among six microsatellite markers. The pattern of allelic variation presented allelic loss in one microsatellite marker direction in T2 of all these five patients, also suggesting that T2 could be derived from T1. However, we did not observe that T1 and T2 showed different allelic patterns for any microsatellite markers in Patient six and ten.

**Table 2:** Allelic variation measured at 6 microsatellite markers for AML patients with regional lymph nodal involvement.

Microsatellite markers		RH68414	SHGC-30026	Csnpnthl1-pcr1–1	GDB:378419	STS-L48546	D16S3394
Patient1	T1	+	++	**+++**	**++++**	+	++
	T2	+	++	**++**	**+++**	+	++
Patient2	T1	+	**+++**	+	+++	+	**+++**
	T2	+	**++**	+	+++	+	**++**
Patient3	T1	+	++	++	**+++++**	+	+++
	T2	+	++	++	**++++**	+	+++
Patient4	T1	+	++	**+++**	**+++++**	+	+++
	T2	+	++	**++**	**+++**	+	+++
Patient5	T1	**+++**	++	++	+++	+	++
	T2	**+**	++	++	+++	+	++
Patient6	T1	+	++	+	+++	+	++
	T2	+	++	+	+++	+	++
Patient7	T1	**++**	++	+++	++++	++++	++
	T2	**+**	++	+++	++++	++++	++
Patient8	T1	+	++	**+++**	+++	+	++
	T2	+	++	**++**	+++	+	++
Patient9	T1	+	**++**	+	+++	+	**++**
	T2	+	**+**	+	+++	+	**+**
Patient10	T1	+	++	+	+++	+	++
	T2	+	++	+	+++	+	++
Patient11	T1	**++**	+	−	−	+	+
	T2	**+**	+	−	−	+	+
Patient12	T1	+	++	**++++**	+++	**+++**	+++
	T2	+	++	**+++**	+++	**++**	+++
Changes/tumor pair		3/12(25%)	2/12(16.7%)	4/12(33.3%)	3/12(25%)	1/12(8.3%)	2/12(16.7%)

*Note: T1, tumor, T2, lymph node; +indicates number of alleles amplified; − indicates a lack of allele amplification.

## Discussion

Although some researchers still regard the renal AML as a hamartoma, a tumor-like malformation formed by aberrantly assembled normal renal tissues, many studies have clearly demonstrated that it is a tumor. Some trials suggested that chromosome X inactivated randomly, gene deficiencies in chromosome band 16p13 and clonal chromosome aberrations in other specified locations may be related to clonal origination of AML^10–12^. Classical AML showed an insidious onset and could be preoperatively differentiated from other renal neoplasms because of its radiographic characteristics^13,14^. If the fat of AMLs was poor, these AMLs were suspected as RCC (renal cell cancer) and could only be identified after surgery^15–17^. Therefore pathological features contributed to the diagnosis^18,19^. Options for treatment of AMLs traditionally included active surveillance for appropriately selected patients^20^, radical or partial nephrectomy^21,22^, selective arterial embolization (SAE)^23,24^ and ablative therapies including cryoablation and radiofrequency ablation (RFA)^25,26^. In the previous reports, the cases with nodal involvement were all treated with radical nephrectomy, besides one received heminephrectomy^3^. While, no report has discussed which surgical procedure (radical nephrectomy or nephron-sparing surgery) is better when lesion volume is relatively small and residual nephron still work.

Regional nodal involvement in a case of renal AML leads to a question of metastasis versus multicentric origin. Especially when the involved lymph node shows the morphologically similarity with the renal AML, it can be impossible to distinguish whether the neoplasms are clonally related (reflecting metastatic disease) or clonally unrelated (representing multicentric nature). As clonal derivation of cells is the hallmark of neoplasia and strongly implicates acquired somatic mutations resulting in a survival advantage to a clonal cell population^27,28^. Thus, clonality analysis could be a powerful tool in determining whether two populations of cells (such as cells from similar or different tumors of the same patient) are genetically similar or different. The methodology based upon p53 mutation status is useful but not practical for broad application in the determination of tumor relatedness^29,30^. Analysis of allelic loss of heterozygosity (LOH) provides another effective method to tackle this problem^31^. Genetic markers are useful in the identification of molecular differences between multiple tumors, however it requires the presence of normal tissue for complete analysis. Considering the germline DNA or normal tissue may not always be available for analysis, especially when the tumor tissue is from biopsy or needle aspirate, which limits the clinical broad application of this method. While the multiply and independently developing neoplasms in an individual patient will possess measurable genomic variations that can be analyzed to generate unique molecular signatures reflecting tumor lineage and relatedness. Allelic variations between neoplasms often reflect the accumulation of differential chromosomal deletion events. Different from the molecular alterations that drive tumorigenesis, these chromosomal deletions are tolerated. Comparison of molecular signatures between multiple tumors from the individual can be used to determine lineage relationship, facilitating the discrimination of second primary cancer versus metastatic disease. Based on this theory, Mercer *et al.*^7^ found that detections of microsatellite alterations and deletion sites in tumor cells DNA could be used as diagnostic and prognostic markers for multiple cancers.

To the authors’ knowledge, this is the first study exploring the clonal origin of AML in kidney and LN. A possible lineage relationship between AML in kidney and LN cannot be excluded. However, in five patients (Patient three, five, seven, eight, and eleven), this relationship was relatively weak and suspicious because of the small number of discordant changes between T1 and T2. The relationship cannot be identified in two patients (Patient six and ten) as no allelic variation was observed in all six microsatellite markers. Patient nine provided a clear example of a patient with possibly clonally-related tumors in whom the evidence suggested that the T2 (lymph node lesion) could be derived from the T1 (kidney tumor). However, the controversial consequences did not happen in all patients.

As this is by no means the gold standard, some limitations of this technology should be mentioned. First, the PCR system is likely to produce non-specific amplification, which could influence the results’ accuracy. Therefore, all microsatellite markers in TSC-2 gene were screened and finally six markers with high reproducibility were remained. The reasons for excluding those markers included their lower specificity, longer amplified fragments, or their band numbers were too many to recognize the differences. Although limited markers were analyzed, the underlying clonal relationship observed might be valid because of the good consistency of results. Meanwhile, there are a large number of microsatellite markers available for any subtype of tumor or disease; thus it is difficult to ensure that whether the suitable microsatellite markers have been chosen to analyze the tumor lineage and relatedness. Furthermore, the differences between researchers could be exaggerated by using the different number or type of microsatellite markers and various PCR systems. Given that there is concern about the effects of intra-tumor heterogeneity on PCR-based analysis, increasing the numbers of microsatellite markers (and observations of allelic variation) might be necessary. But it remains unknown how many microsatellite markers are sufficient for analysis.

In summary, polymorphic microsatellite markers analyses are useful to distinguish the lineage relationship between tumors and the enlargement lymph nodes. Furthermore, with this technique, there is a possible lineage relationship between AMLs in kidney and in LN although AMLs were considered as a benign tumor. Thus when enlarged lymph nodes were found in images, active surveillance should be taken into consider; if enlarged lymph nodes were found intraoperatively, local lymph node/nodes resection might be necessary to demonstrate their pathological nature, which contributes to make the corresponding follow-up plan.
