# Author Correction: Microsatellite analysis for differentiating the origin of renal angiomyolipoma and involved regional lymph node

**DOI:** 10.1038/s41598-018-33870-5

**Published:** 2018-10-17

**Authors:** Ping Tan, Huan Xu, Yong Jiang, Lu Yang, Yan Zou, Liangren Liu, Nian Liu, Dehong Cao, Yu Fan, Qiyuan Li, Qiang Wei

**Affiliations:** 1 Department of Pathology, West China Hospital, Sichuan University, Chengdu, Sichuan People’s Republic of China; 2 Department of Urology, West China Hospital, Sichuan University, Chengdu, Sichuan People’s Republic of China; 3 Institute of Urology, West China Hospital, Sichuan University, Chengdu, Sichuan People’s Republic of China

Correction to: *Scientific Reports* 10.1038/s41598-017-00460-w, published online 23 March 2017

The original PDF version of this Article contained an error in the order of corresponding authors. This has now been corrected in the PDF version of this Article

